# Structural and Electrical Analysis of Crystalline Silicon Solar Cells: The Role of Busbar Geometry in First-Generation PV Technology

**DOI:** 10.3390/ma18214979

**Published:** 2025-10-31

**Authors:** Małgorzata Monika Musztyfaga-Staszuk, Claudio Mele

**Affiliations:** 1 Materials Investigating Laboratory, Faculty of Mechanical Technology, Silesian University of Technology, Konarskiego 18A Street, 44-100 Gliwice, Poland; 2 Dipartimento di Ingegneria dell’Innovazione, Università del Salento, Via Monteroni, 73100 Lecce, Italy; claudio.mele@unisalento.it

**Keywords:** crystalline solar cells, different generations of solar cells, electrical, structural properties

## Abstract

This study focuses on first-generation crystalline silicon photovoltaic (PV) cells, which remain the core of the PV industry. It outlines the structure and operation of single-junction cells, distinguishing between monocrystalline and polycrystalline technologies. A literature review was conducted using databases such as Web of Science and Scopus to identify research trends and inform future research directions. PV cell classification by generation is also presented based on production methods and materials. The experimental section includes both electrical and structural characterisation of crystalline silicon solar cells, with particular emphasis on the influence of the number and geometry of front-side busbars on metal-semiconductor contact resistance and electrical properties. Additionally, the paper highlights the use of dedicated laboratory equipment—such as a solar simulator (for determining photovoltaic cell parameters from current-voltage characteristics) and Corescan equipment (for determining layer parameters using the single-tip probe method)—in evaluating PV cell properties. This equipment is part of the Photovoltaics and Electrical Properties Laboratory at the Silesian University of Technology. The findings demonstrate clear structural correlations that can contribute to optimising the performance and longevity of silicon-based PV cells.

## 1. Introduction

The photovoltaic industry represents a contemporary manufacturing sector that heavily depends on automation and the implementation of advanced technologies and technical solutions. As a result, photovoltaics is gaining increasing popularity and is regarded as a crucial component of the energy transition in numerous countries worldwide. Photovoltaics (PV) is a branch of science and technology focused on the conversion of solar energy into usable electrical energy. This process entails harnessing electrical energy from solar radiation through various photovoltaic components, including photovoltaic cells that can be assembled into photovoltaic modules. Recent technological advances have improved solar panel efficiency and reduced production costs, making solar energy more accessible to both businesses and households [1,2,3,4,5,6].

The primary objective of this work is its educational value, which can contribute to the popularisation of knowledge among a broad audience. An additional advantage of the study is the organised presentation of definitions of basic physical quantities and parameters that characterise photovoltaic cells. The work also includes an overview of first-generation photovoltaic cells based on crystalline silicon, which form the foundation and dominant segment of the photovoltaic industry. Their operating principles, types, advantages, and technological challenges are discussed, along with the basic structural and measurement characterisation, with particular emphasis on the electrical properties of solar cells that differ in the shape and size of front-side metallization.

### 1.1. Generations of Photovoltaic Solar Cells

Solar cells can be classified in several ways, depending on various criteria, such as materials, production technology, and application. Considering the production process technology and the materials used, photovoltaic cells are divided into generations (the first (I), the second (II), the third(III), the fourth (IV) [7,8,9].

The first generation of photovoltaic technology employs traditional silicon cells, both monocrystalline and polycrystalline, primarily produced by companies in China and partially in Europe. First-generation silicon cells represent a milestone in the development of photovoltaic technology. Their history dates back to the early days of solar panel development, and their unique properties and potential have contributed to the rapid growth of the renewable energy market [10].

Types of First-Generation Silicon Cells [10,11]:

- Monocrystalline Cells (mono-Si) are characterised by high efficiencies, often over 20%, with longer lifespans and better performance, which leads to potential savings. However, they are typically more expensive to produce and have a smooth surface. The production process involves growing crystals and slicing them into thin wafers.
- Polycrystalline Cells (multi-Si) have somewhat lower efficiency (around 15–17%, below 20%) but are cheaper to produce. Their surface typically has a slightly matte appearance and is often grey or blue. Arising from the production method, these cells often exhibit greater variations in size and shape. The production process of these solar cells involves melting silicon and cooling it in moulds, which leads to the creation of a material composed of many crystals.

The second generation of photovoltaic cells includes cells manufactured by depositing thin layers of semiconductor materials on various substrates. The second generation of photovoltaic cells represents a significant advancement in photovoltaics but also presents challenges due to the use of environmentally harmful materials and low efficiency in converting solar energy to electricity [10].

Types of Second-Generation Solar Cells [10,11,12]:

- Amorphous (a-Si)—They generally have lower conversion efficiency (around 6–10%) than traditional crystalline cells but perform better in low-light conditions and are less sensitive to high temperatures, making them suitable for areas with limited sunlight. These cells are cheaper to produce, making them more affordable for specific applications (e.g., sundials). However, they may degrade more quickly over time, leading to a decline in efficiency. This type of silicon lacks a regular crystalline structure, with its atoms arranged randomly. This irregular arrangement influences the material’s electrical and optical properties.
- Copper–Indium–Gallium-Selenide (CIGS)—CIGS cells consist of a thin layer of active material on a substrate, typically glass, stainless steel, or plastic, resulting in lightweight and flexible cells. They achieve a relatively high energy conversion efficiency of up to 23% in laboratory settings, making them among the most efficient thin-film cells. Recent production advancements have further improved their efficiency, and their compatibility with flexible substrates opens up various application possibilities. Cadmium Telluride (CdTe). The materials used in CIGS cell production, such as indium and selenium, may raise environmental concerns. However, this technology can promote renewable energy development and reduce CO_2_ emissions compared to fossil fuel-based energy methods.
- Cadmium Telluride (CdTe)—CdTe cells achieve energy conversion efficiencies of about 15–22%, making them competitive with other photovoltaic types. They are produced through a chemical deposition method, resulting in thin-film, semi-transparent structures. These cells are commonly used in large-scale photovoltaic farms and smaller home installations. However, their disposal poses challenges due to toxic materials, necessitating the development of effective recycling processes. CdTe is a cost-effective material, and the production process for CdTe cells is more affordable than that of crystalline cells. This affordability makes them appealing for commercial applications.

The third generation of photovoltaic cells comprises dye-sensitised, organic, and perovskite cells. Ongoing research focuses on improving their stability and durability while maintaining low production costs—a challenge for scientists [10].

Types of Third-Generation Cells [11,12,13,14,15]:

- Dye-Sensitised Solar Cells (DSSC) utilise organic dyes, enabling production in various colours for architectural applications and enhancing building aesthetics. They have lower production costs than traditional silicon cells and can be applied to different substrates, including flexible materials. However, they have lower energy conversion efficiency (from 7 to 11%). However, some components, such as electrolytes, may degrade over time and are sensitive to atmospheric conditions, particularly humidity and temperature.
- Organic cells contain heavy metals and use organic compounds as electrodes or electrolytes, making them perceived as more environmentally friendly. These cells can be produced in lightweight and flexible forms for use in portable devices and modern architecture. However, challenges remain, including the degradation of organic materials, longevity, and lower conductivity compared to traditional materials, which continue to be areas of ongoing research.
- Perovskite achieves conversion efficiencies over 25% in lab tests and is relatively inexpensive to produce compared to traditional silicon solar cells. However, challenges remain regarding long-term stability and resistance to weather conditions like humidity and temperature. Their versatility allows for use on various substrates, making them suitable for flexible and lightweight photovoltaic panels.

Heterojunction photovoltaic cells (HJT) are considered the next generation of photovoltaic cells. With a conversion efficiency of up to 24% for a single solar cell, they compare favourably to standard polycrystalline and monocrystalline cells with an n-p junction. Multi-junction photovoltaic cells use multiple semiconductor layers to absorb various ranges of solar radiation, with each layer optimised for specific wavelengths. These cells feature a combination of monocrystalline and thin-film silicon layers, enabling high solar energy conversion efficiency. A passivating layer (e.g., a-Si) reduces surface recombination [16].

The global photovoltaic market is expected to experience growth in the coming years, influenced by various factors such as energy policies, technological advancements, fluctuations in raw material prices, and increasing public interest in renewable energy.

The published report [10] indicates that the estimated production of modules in 2023 is projected to range between 460 and 502 GW [10]. Solar cells can be classified in several ways, depending on various criteria, such as materials, production technology, and application. Taking into account the production process technology and the materials used, photovoltaic cells are divided into generations (the first (I), the second (II), the third (III), the fourth (IV) [7,8].

The 2023 report from the International Energy Agency Photovoltaic Power Systems Programme (IEA-PVPS) estimates the installed capacity at 1581 GWp, while the International Renewable Energy Agency (IRENA) cites a figure of 1412 GWp [10]. Analysing this data, it is evident that China remains the leading country in terms of power generated by photovoltaic installations.

### 1.2. Construction and Operating Principle of a Single-Junction Silicon Photovoltaic Cell

**Figure 1:**
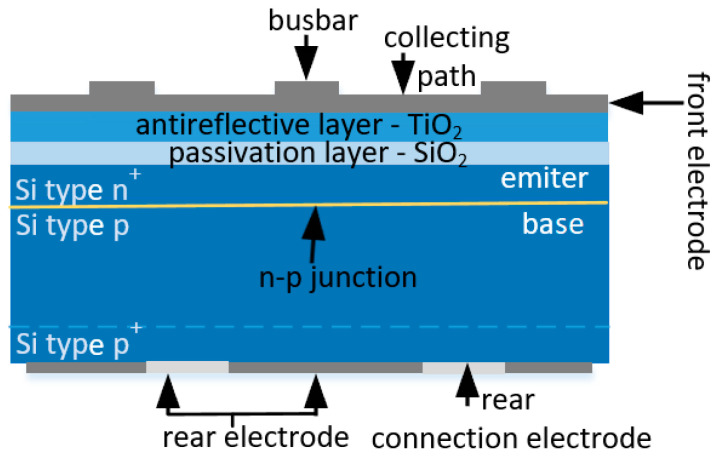
Cross-sectional view of a single-junction silicon photovoltaic cell featuring a back field that reflects carriers, a passivation layer, and an anti-reflective coating [8,21].

Most photovoltaic cells currently available on the market are made using monocrystalline silicon grown by the Czochralski method (Cz-Si), doped with boron (p-type silicon). The fundamental element of the cell discussed is the n^+^-p junction formed through the diffusion process of phosphorus (an n-type dopant) into p-type silicon. The heavily doped, surface n+ layer acts as the emitter layer of the photovoltaic cell. The p-type silicon region is called the base layer. The n^+^-p junction should be formed at an optimal distance from the surface of the cell (approximately 0.5 µm). Figure 1 illustrates the structure of a single-junction crystalline photovoltaic cell, which comprises several layers: an emitter layer (n-type Si), a base layer (p-type Si), a highly doped silicon layer (Si p^+^) that creates the reversing field, and front layers like a passivation layer and anti-reflective coating (ARC). The front and rear electrodes collect the generated carriers, and optimal formation of these layers greatly influences the efficiency of electromagnetic radiation absorption [14,15,16]. Most global manufacturers of silicon-based photovoltaic cells utilise a multi-step process that includes chemical etching, surface texturing, donor doping for n^+^-p junction formation, applying passivation and anti-reflective layers, screen printing front and rear electrodes, and thermal annealing for electrode formation [17,18,19,20]. Mass production of photovoltaic cells frequently employs hybrid technology, with screen printing as a crucial component for forming the front and rear electrodes. This method can also create an n^+^ emitter layer and a heavily doped p^+^ layer, generating a back surface field (BSF) using specialised pastes [11,21,22].

To transfer charge carriers generated and separated in the solar cell, metal electrodes are used. The main challenge in the mass production of solar cells is finding techniques that would reduce manufacturing costs. One such technique is screen printing, which allows for the production of layers with various properties. “After printing, the final properties of the layer (electrode) are achieved after drying and co-firing in a tunnel furnace at a specific temperature and for a specific duration”.

Selecting materials for electrodes in silicon photovoltaic cells requires extensive knowledge of both theory and practice. Numerous publications cover material selection and fabrication techniques. The collection electrode design may feature one or more busbars, depending on cell dimensions [22,23,24].

In a silicon single-junction photovoltaic cell, solar radiation energy is converted into usable electrical energy through two physical phenomena:

1. The internal photoelectric effect,
2. The transport of generated charge carriers within the cell structure, which occurs due to the influence of the electric field and diffusion driven by the concentration gradient of the carriers.

The photovoltaic effect, discovered by E. Becquerel in 1839, initiated research on the photoelectric effect, continued by his family. The first monocrystalline silicon photovoltaic cell was patented by R. Ohm in 1941; however, it had low efficiency. It was not until 1954 that G. Pearson, D. Chapin, and C. Fuller at Bell Laboratories created the first silicon photovoltaic cell capable of generating measurable current. Regardless of the type of semiconductor—whether monocrystalline or polycrystalline silicon-exposing the material to light can induce the photovoltaic effect, although its intensity may vary depending on the wavelength of the light [25,26,27].

When the semiconductor is illuminated, the concentration of electrons and holes changes according to Equations (1) and (2) [28,29].

(1) $$n\;=\;n_{i}\;+\;\Delta_{n}\;a,$$

(2) $$p\;=\;p_{i}\;+\;\Delta_{p}\;n.$$

where Δ_n_—concentration of photogenerated electrons; Δ_p_—concentration of photogenerated holes; n_i_—electron concentration in an intrinsic semiconductor; pi—hole concentration in an intrinsic semiconductor

For the energy of absorbed radiation to excite an electron from the valence band to the conduction band, it must meet the condition specified in Equation (3). The energy of radiation quanta must be greater than the width of the energy gap of the semiconductor W_g_ [28,29]:

(3) $$W_{g}\leq h\cdot v$$

Therefore, photogeneration in a semiconductor with an energy gap W_g_ will be caused by radiation with a wavelength shorter than λ_max_:

(4) $$\lambda_{\max}<\frac{h\cdot c}{W_{g}}$$

where λ_max_—long-term limit of photogeneration

Each absorption of a photon by an electron in the valence band moves it to the conduction band, creating a hole in the valence band. This process increases the carrier concentration, especially in the n^+^-p junction area, resulting in free electrons in the conduction band and holes in the valence band. Consequently, photogeneration enhances both electron and hole concentrations, affecting the electrical properties of the semiconductor.

In a non-illuminated intrinsic semiconductor, the concentration of charge carriers—electrons and holes—generated thermally depends on temperature and material type. As the temperature rises, the number of charge carriers increases, enhancing the material’s electrical conductivity.

### 1.3. Publication Trends in Monocrystalline and Polycrystalline Silicon Solar Cells (Supplementary Information)

Statistical data from electronic databases can cover various areas of science. This paper discusses issues specifically related to materials science, with an emphasis on photovoltaics. International interdisciplinary databases, such as Scopus and the Web of Science Core Collection, focus on indexing high-quality publications. Publishers analyse the scientific quality of these publications and calculate metrics such as Impact Factor and CiteScore to evaluate journals. Utilising electronic databases like Scopus and Web of Science offers numerous benefits for scientists, research institutions, and students at both high school and university levels. One of the key advantages is the ability to select appropriate journals for research publication, which facilitates the targeting of the right audience. Users can also find potential research partners based on their publications and identify research trends, which helps formulate hypotheses and plan future projects [30].

**Figure 2:**
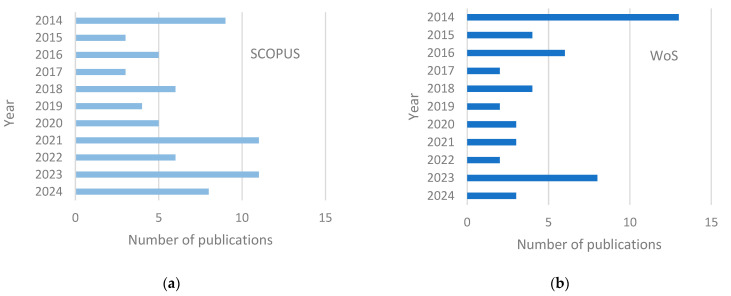

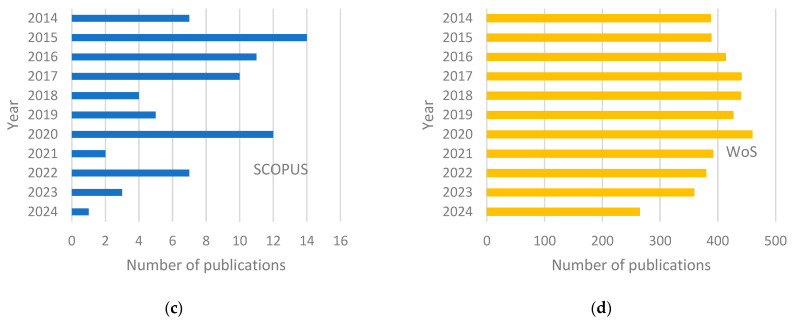
The number of publications each year related to photovoltaic cells ((**a**,**b**)—monocrystalline, (**c**,**d**)—polycrystalline) as reported by (**a**,**c**) Scopus and (**b**,**d**) Web of Science databases.

Between 2014 and 2024, the Scopus database recorded 71 publications, while the WoS database recorded 50 publications, all of which were searched using the term “monocrystalline solar cells” (Figure 2a,b). Based on the data analysis conducted for this study, it can be concluded that the most significant number of publications in the Scopus database (11 each) was recorded in 2021 and 2023, while the WoS database saw its highest number of publications (13) in 2014. The diagrams presented in Figure 1 indicate that in the last two years, a similar upward trend in the number of published works can be observed in both the Scopus and WoS databases.

Between 2014 and 2024, the Scopus database recorded 76 publications, while the WoS database recorded 4344 publications, all of which were searched using the term “polycrystalline solar cells” (Figure 2c,d). Based on the data analysis, it can be concluded that the WoS database had a significantly larger number of publications than the Scopus database. Scopus recorded its highest number of publications in 2015, with 14 papers. An upward trend was observed in Scopus between 2015 and 2017, as well as between 2020 and 2022. The highest number of publications in the WoS database was recorded in 2020, with 459 publications. Before this year, an upward trend in the number of published papers was observed, but after 2020, this trend began to decline.

The 2024 report [10] shows a clear dominance of crystalline silicon-based photovoltaic cells. This dominance is due mainly to the abundant availability of silica, the primary feedstock. Standardised production techniques for monocrystalline (Cz-Si) and multicrystalline silicon (mc-Si) have facilitated automation in manufacturing photovoltaic cells and modules. Additionally, there is a comprehensive database of diagnostic and research data on silicon, supported by a well-established electronics industry.

## 2. Materials

**Figure 3:**
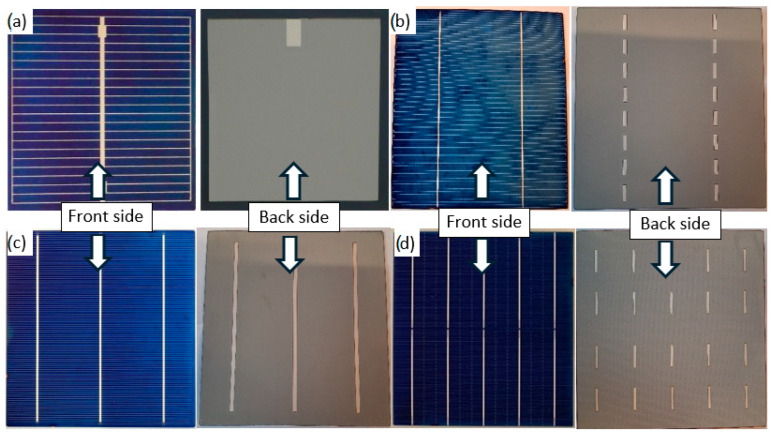
View of investigated silicon solar cells with: (**a**) one busbar, (**b**) 2 busbars, (**c**) 3 busbars, (**d**) 5 busbars.

Figure 3 presents the multicrystalline silicon solar cells (with a varying number of busbars) investigated in this paper. A single-busbar cell was fabricated under laboratory conditions, whereas the remaining cells were commercial units procured from various manufacturers (Table 1).

Below is an example of a technological process for fabricating a solar cell with a single busbar under laboratory conditions.

The technological process of solar cell fabrication involves a series of operations aimed at preparing and forming the functional semiconductor structure. The first step is the degreasing of the wafer’s surface to remove organic contaminants. This is followed by the removal of the highly defective surface layer and surface texturisation to improve light trapping and reduce reflection. The next stage is wafer surface cleaning, which includes the removal of organic and metallic contaminants, as well as natural oxide layers. Subsequently, the p–n junction is formed through a diffusion process. After diffusion, the donor dopant is removed from the wafer edges, and the glass layer formed during diffusion is eliminated. The process continues with surface passivation followed by the application of an anti-reflective coating. Front and rear electrodes are then applied using screen printing, and the final step involves the firing of the front electrodes to complete the cell fabrication process.

**Figure 4:**
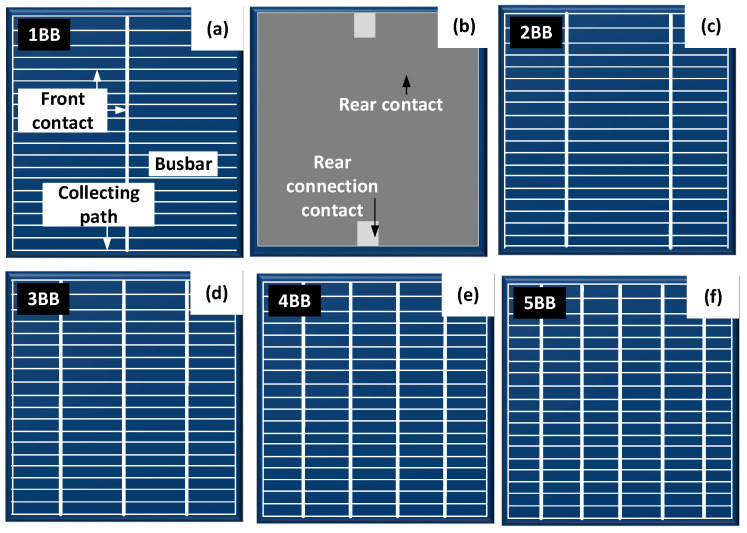
View of (**a**) the front and (**b**) the rear electrode of a photovoltaic cell with one busbar. View with subsequent front busbars of the solar cell (**c**) 2, (**d**) 3, (**e**) 4, (**f**) 5.

Figure 4 shows examples of front and rear electrode shapes in commercial silicon photovoltaic cells.

## 3. Methodology

The structure was analysed by scanning electron microscopy (SEM, ZEISS, Oberkochen, Germany), for surface and cross-sectional topography of the front electrode and silicon substrate, with energy dispersive X-ray spectroscopy (EDS) used for chemical composition analysis of selected areas.

**Figure 5:**
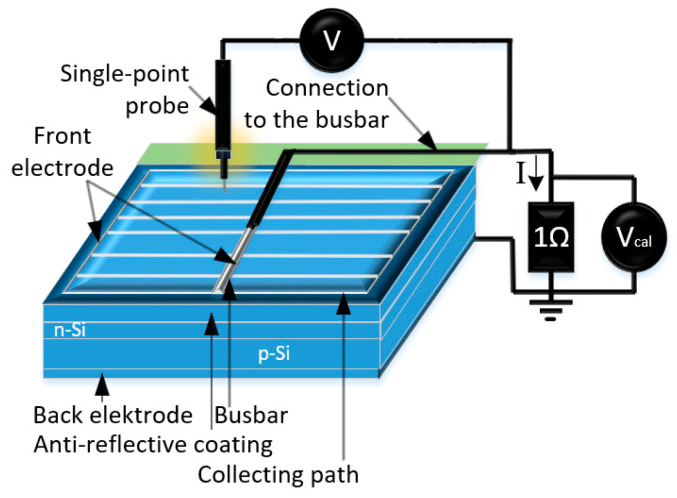
Measurement schematic using the Potential Difference (PD) method with a probe [31].

The Corescan device, developed by SunLab and manufactured by the Dutch company Mechatronics (ZEISS, Germany), was used in this study in Core Scan mode (Contact Resistance Scanner) to determine the resistivity (ρ) and contact resistance (R_c_) of the emitter layer in the analysed solar cells. This workstation, equipped with a single-point probe and dedicated software, allows for precise measurement of local properties of solar cells. For nearly two decades, Mechatronics has been producing and distributing this commercial research tool under the brand name “Corescan,” primarily for scientific analysis of photovoltaic devices [28]. The Potential Difference (PD) method is used with a single-point probe in Core scan mode to analyze selected areas of a solar cell (Figure 5). The measurement begins by setting the current flowing through a 1Ω resistor, which is controlled via the calibration voltage V_cal_. The current is generated by local illumination, with the illuminated spot having a diameter of 10 mm. During the measurement, the single-blade probe scans the front surface of the cell, recording voltage variations. The probe can move in one or both directions, parallel to the front electrode busbar. In the single-direction mode, the surface is scratched only during the forward pass, with the probe lifted during the return. In the two-direction mode, the surface is scratched during both forward and return movements.

Measurement Conditions:

In the “SCAN SETTINGS” tab, scanning parameters are set according to the measurement type, which can include the following:

- Contact resistance Rc and resistivity ρ (Core Scan);
- Open-circuit voltage U_oc_ (U_oc_ Scan);
- Voltage U_R_ (Shunt Scan);
- Photogenerated current density (LBIC Scan).

Accurate dimensions of the measured object (e.g., a solar cell) must be entered with 0.1 mm precision to ensure accurate calculation of the scan area. The software automatically applies a 1 mm safety margin around the sample edges. Scan time depends on the scan line spacing, scan speed, and direction (single or bidirectional scanning). These parameters can be checked and adjusted before starting the measurement. Settings and results can be saved to a file and reloaded for future use.

Efficiency and fill factor were determined using a setup that included the SS150AAA solar simulator from Photo Emission Tech (Moorpark, CA, USA).

## 4. Results and Discussion

**Figure 6:**
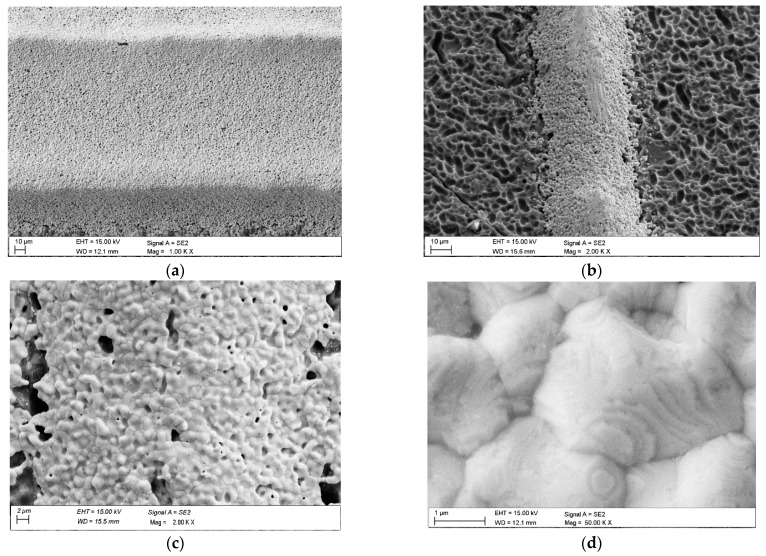
Surface topography of front side electrode (SEM): (**a**) 1 BB, (**b**) 2 BB, (**c**) 3 BB, (**d**) 5 BB.

Figure 6 shows the textured surface of silicon solar cells. Scanning electron microscope (SEM) measurements in subfigures (a) and (d) show that the pyramids formed by this process have an average height of about 3 µm. The pattern visible across the figure indicates that this texture was probably created by treating the silicon in an acidic solution with a specific chemical mix. However, the results in subfigures a–d suggest that a similar etching procedure was achieved using two different solutions. Metallographic observations conducted with a scanning electron microscope (SEM) show that the front electrode morphology, made from a commercial silver paste, is porous. 

**Figure 7:**
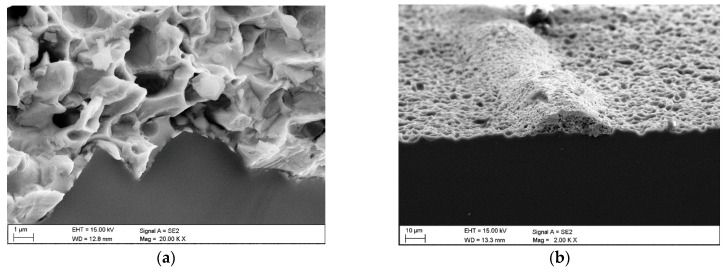

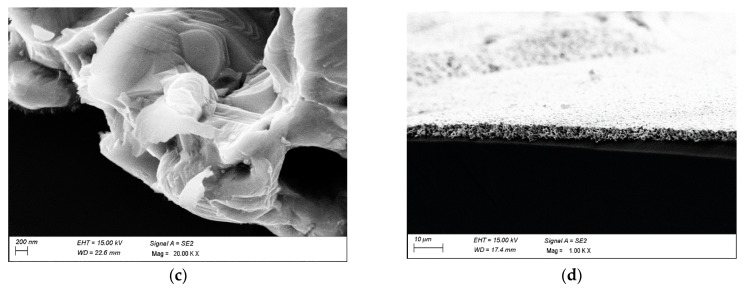
SEM cross-sectional view of the front electrode contact area with the silicon substrate in solar cells with: (**a**) 1 BB, (**b**) 2 BB, (**c**) 3 BB, (**d**) 5 BB.

Figure 7 presents a cross-section of the examined samples. Metallographic observations conducted using SEM show that the cross-section of the investigated solar cells adheres well to the silicon substrate.

**Figure 8:**
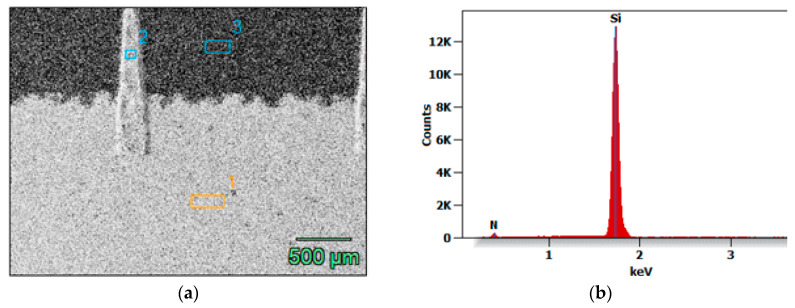
(**a**) Energy-dispersive X-ray spectroscopy (EDS) analysis of selected micro-areas of the solar cell (1—busbar, 2—finger, 3—substrate); (**b**) EDS spectrum from micro-area 1.

The EDS results for the chemical composition of selected fragments of the 5BB solar cell are provided in Table 2 and Figure 8.

The results indicate that Area 1 is primarily silver. While silver also dominates Area 2, it contains traces of silicon. In contrast, Area 3 consists mainly of silicon with minor nitrogen content, likely introduced by surface passivation via an inert N_2_ gas stream.

**Figure 9:**
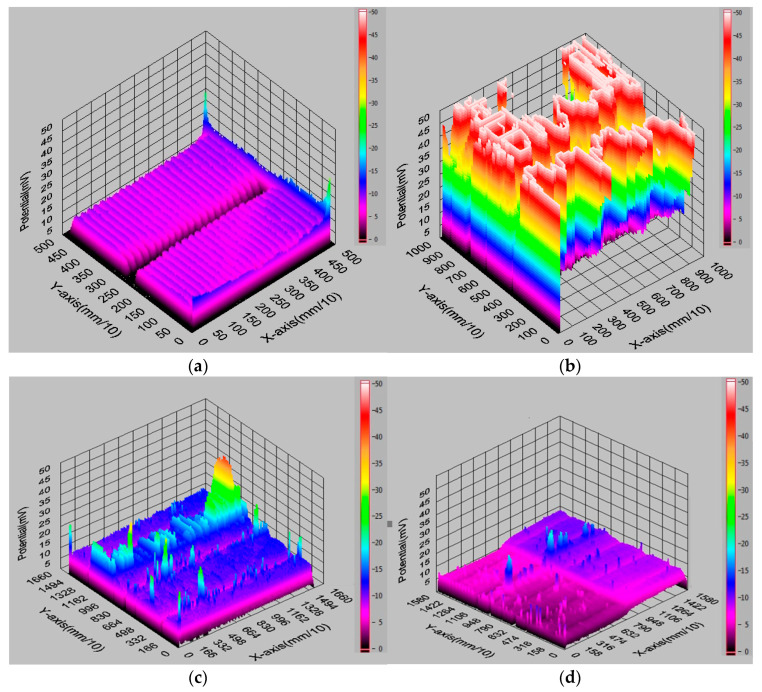
A view of the measurement of selected electrical property parameters of solar cells: (**a**) 1BB, (**b**) 2BB, (**c**) 3BB, (**d**) 5BB; obtained in Core scan mode on the Corescan device.

**Figure 10:**
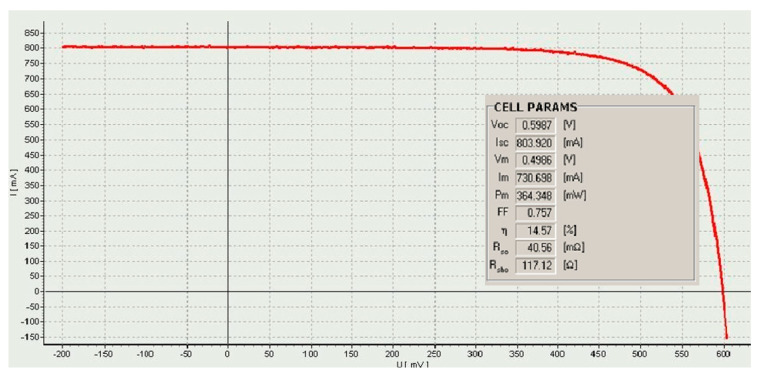
Measured I–V characteristic of a one-busbar solar cell (an example).

Figure 9 and Table 3 present the measurement results obtained using the single-probe method. Figure 10 shows selected electrical parameters of the front metallisation of solar cells. Table 2 presents an example of a measurement conducted at a chosen point on a solar cell at a specific voltage.

For the Corescan device, a current density of 30 mA/cm^2^ was applied, which corresponds to a current value of 750 mA from a cell with an area of 25 cm^2^. In the Corescan device, the current is generated by a small light beam and flows while the photovoltaic cell is short-circuited by an external load (a resistor). Based on the 3D graphical analysis (Figure 9b,c), it can be stated that the brighter areas (potential > 25 V) observed on the surface of the investigated samples are characterised by a significantly higher potential and higher resistance at the junction. They also confirm a high non-uniformity of the (R_cont_) parameter, which likely results from temperature differences between the scanned stripes on the front surface of the tested sample. The scanned 3D images, primarily in Figure 9a,d, show a high uniformity of the (R_cont_) parameter. Figure 10 shows an example measurement of electrical parameters from a station equipped with a solar simulator for a selected solar cell.

Table 4 and Figure 10 contain the calculated values of electrical parameters from additional software installed on the computer included in the I-V measurement station [29]. Figure 10 shows an example measurement of electrical parameters from a station equipped with a solar simulator for a selected cell.

Research indicates that increasing the number of busbars reduces a solar cell’s series resistance, thereby enhancing its maximum power, conversion efficiency, and fill factor.

The highest efficiency was achieved for the solar cell with 5BB, and the lowest for the one with a single busbar with 1BB. The differences in the results are mainly due to the manufacturing process used to produce them. The measurement results in Table 4 show a clear trend: series resistance values decrease as the number of busbars increases. These results confirm the intuitive understanding that adding more busbars reduces the series resistance of a solar cell.

## 5. Conclusions

The study found that increasing the number of busbars in silicon solar cells significantly reduces series resistance, thereby improving power output, efficiency, and fill factor. The front electrode made from silver paste has a porous structure that adheres well to the silicon substrate. Elemental analysis confirmed the expected material composition, including traces of nitrogen from surface passivation. Using the Corescan device, detailed maps of contact resistance were obtained without special sample preparation, revealing areas of higher resistance linked to temperature variations. Overall, adding more busbars enhances solar cell performance by lowering resistance and improving electrical characteristics.

Despite the dynamic development of new technologies, first-generation solar cells remain the foundation of the global photovoltaic market. Their proven reliability and high efficiency ensure that they will continue to be the primary tool in the fight for clean energy for many years to come. Their future lies not in revolution, but in continuous, evolutionary improvement. Key development directions include further optimisation of the production process and cost reduction, driven by advancements in PERC, HJT, and TOPCon technologies that enhance their efficiency from within. Another promising path is the development of tandem cells, which combine a traditional silicon layer (1st gen.) with a perovskite layer (3rd gen.) to overcome inherent efficiency limits and create a next-generation hybrid technology.

## Figures and Tables

**Table 1:** Selected basic properties of solar cells (p-type silicon doped with boron).

Number of Busbars in a Solar Cell	Area	Thickness
One	5 cm × 5 cm ± 0.25 mm	200 ± 30 μm
Two	10 cm × 10 cm ± 0.25 mm	150 ± 30 μm
Three	16.6 cm × 16.6 cm ± 0.25 mm	175 ± 30 μm
Five	15.8 cm × 15.8 cm ± 0.25 mm	180 ± 30 μm

**Table 2:** Selected Chemical composition of selected locations within the solar cell (where 1—busbar, 2—collecting finger, x3—substrate).

The Area	Weight Fraction of Elements,%		
	N	Si	Ag
1			100.0
2		0.8	99.2
3	16.8	83.2	

**Table 3:** Measurements of contact resistance and emitter layer resistivity in solar cells (where J_sc_—current density illuminated by the scanning probe, ρ—resistivity, R_cont_—contact resistance of the emitter layer, U_ce_—voltage on the collecting track measured with a metal probe in direct contact with its surface.

Number of Busbars in a Solar Cell	J_sc_, mA/cm^2^	U_ce_, mV	R_cont_, mΩcm^2^	ρ, Ωcm
1BB	30	8.8	26	2.6
		9.3	28	2.8
		18.6	56	5.6
2BB		11.7	84	7.0
		18.6	134	11.1
		30.8	221	18.5
3BB		7.3	13	1.1
		14.2	25	2.1
		45.9	83	6.9
5BB		22.5	162	13.5
		36.1	260	21.7
		55.7	401	33.4

**Table 4:** Electrical parameters of solar cells (where U_oc_—open-circuit voltage, I_sc_—short-circuit current, U_m_—voltage at maximum power), I_m_—current at maximum power, Pm—maximum power, FF—fill factor, E_ff_—conversion efficiency) [4].

Number of Busbars in a Solar Cell	Parameters					
	I_sc_, mA	U_oc_, mV	P_m_, mW	FF	E_ff_, %	R_so_@U_oc_, mΩ
1BB	804.9	598.7	364.3	0.76	14.57	40.56
2BB	7969.0	672.1	3133.2	0.59	15.54	18.3
3BB	8207.4	668.9	3315.9	0.60	16.09	15.6
5BB	8076.2	670.9	3665.6	0.68	17.91	10.4

## Data Availability

The original contributions presented in this study are included in the article. Further inquiries can be directed to the corresponding author.
